# Structural and Biophysical Characterization of the Cytoplasmic Domains of Human BAP29 and BAP31

**DOI:** 10.1371/journal.pone.0071111

**Published:** 2013-08-13

**Authors:** Esben M. Quistgaard, Christian Löw, Per Moberg, Fatma Guettou, Karthik Maddi, Pär Nordlund

**Affiliations:** 1 Department of Medical Biochemistry and Biophysics, Karolinska Institutet, Stockholm, Sweden; 2 School of Biological Sciences, Nanyang Technological University, Singapore, Singapore; Instituto de Tecnologica Química e Biológica, UNL, Portugal

## Abstract

Two members of the B-cell associated 31 (BAP31) family are found in humans; BAP29 and BAP31. These are ubiquitously expressed receptors residing in the endoplasmic reticulum. BAP31 functions in sorting of membrane proteins and in caspase-8 mediated apoptosis, while BAP29 appears to mainly corroborate with BAP31 in sorting. The N-terminal half of these proteins is membrane-bound while the C-terminal half is cytoplasmic. The latter include the so called variant of death effector domain (vDED), which shares weak sequence homology with DED domains. Here we present two structures of BAP31 vDED determined from a single and a twinned crystal, grown at pH 8.0 and pH 4.2, respectively. These structures show that BAP31 vDED forms a dimeric parallel coiled coil with no structural similarity to DED domains. Solution studies support this conclusion and strongly suggest that an additional α-helical domain is present in the C-terminal cytoplasmic region, probably forming a second coiled coil. The thermal stability of BAP31 vDED is quite modest at neutral pH, suggesting that it may assemble in a dynamic fashion *in vivo*. Surprisingly, BAP29 vDED is partially unfolded at pH 7, while a coiled coil is formed at pH 4.2 *in vitro*. It is however likely that folding of the domain is triggered by other factors than low pH *in vivo*. We found no evidence for direct interaction of the cytoplasmic domains of BAP29 and BAP31.

## Introduction

**Figure 1:**
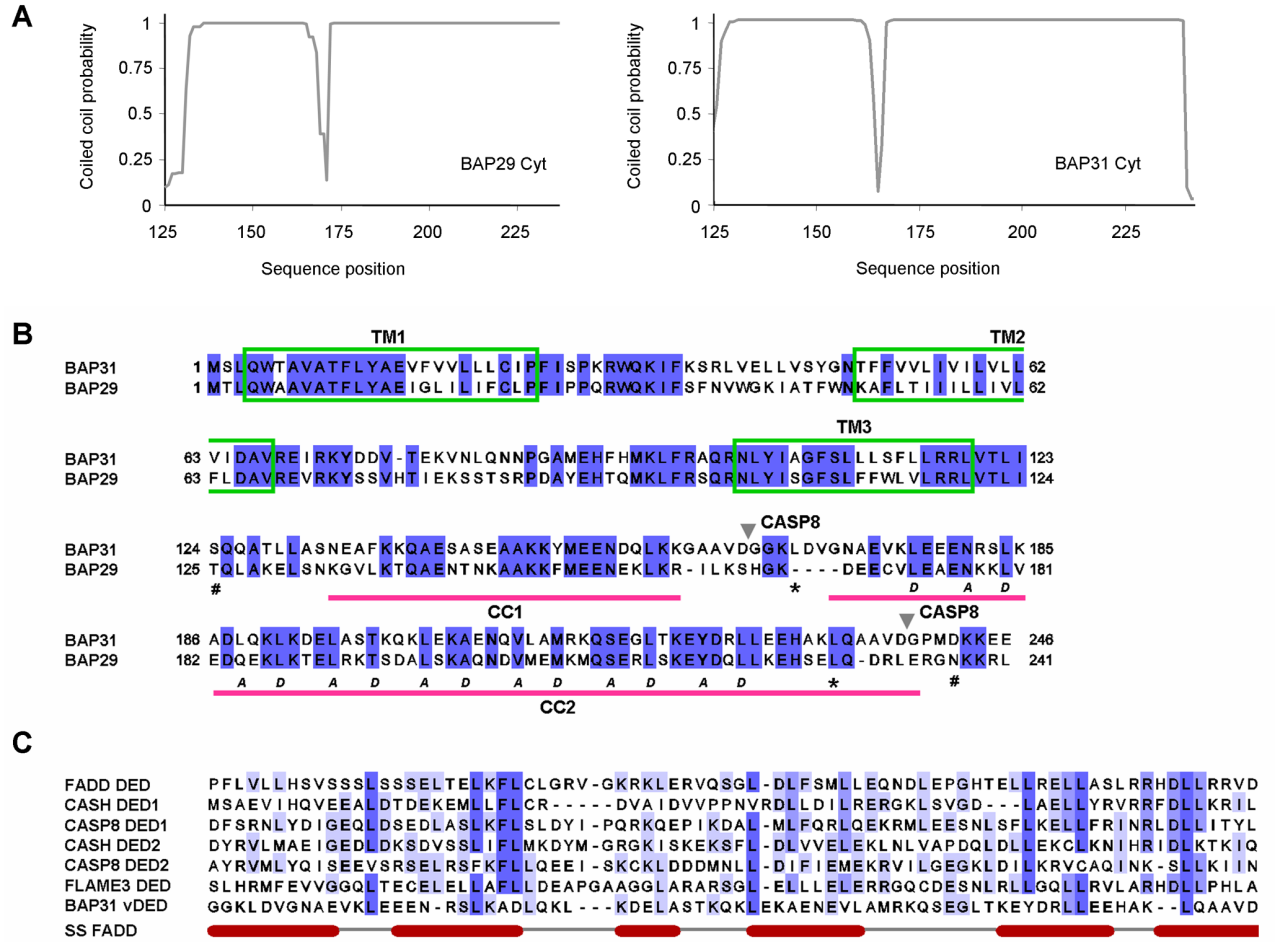
BAP29 and BAP31 coiled coil predictions and sequence alignments. (**a**) Coiled coil prediction. The prediction was carried out on the full-length C-terminal cytoplasmic domains of BAP29 (left) and BAP31 (right) using the Coils server (window size; 21). Similar results were obtained with the Paircoil2 server, except that the N-terminal most coiled coil scored under the default threshold in the case of BAP29 (not shown). (**b**) Global pairwise sequence alignment of human BAP29 and BAP31. Residues shaded blue are identical. For BAP31, the predicted transmembrane helices are boxed in green and the two coiled coils predicted using the Coils server are labeled CC1 and CC2 and marked with pink bars (a score of 0.9 was used as cut-off). Grey triangles denote the caspase-8 cleavage sites in BAP31, (#) symbols denote start and end residues of the full-length C-terminal cytoplasmic regions used in this study, and (*) symbols denote start and end of the vDED constructs. The ‘a’ and ‘d’ positions of the coiled coil heptad repeats of BAP31 vDED are labeled according to the crystal structure. (**c**) Multiple BAP31 vDED:DED sequence alignment. BAP31 vDED (here defined as the region between the two caspase-8 cleavage sites) was aligned with six different human DED domains; DED1 and DED2 from caspase-8 (UniProt: Q14790) and from CASH (UniProt: O15519), and the single DED domains of FADD (UniProt: Q13158) and FLAME3 (UniProt: Q8WXF8). The alignment is colored in shades of blue according to sequence identities and annotated with the secondary structure of FADD. BAP31 vDED, shares 10–18% sequence similarity with caspase-8 DED1 (15%), caspase-8 DED2 (18%), FADD DED (18%), CASH DED1 (11%), CASH DED2 (10%) and FLAME3 DED (16%).

BAP29 and BAP31 are two homologous membrane proteins (∼50% sequence identity in humans) that reside in the endoplasmic reticulum (ER) [1]. They both consist of an N-terminal membrane-bound domain with three predicted transmembrane helices, and a C-terminal cytoplasmic region, which encompass ∼50% of the protein and is predicted to form a coiled coil [2], [3], or more likely two coiled coils (Fig. 1a–b). However, the second and longest of these putative coiled coils also shares weak sequence similarity with the small six-helical DED domains, suggesting that it may instead adopt a DED fold (Fig. 1c) [4]. Due to this homology it has been named ‘variant of DED’ (vDED). Curiously, we find that many of the highly conserved residues are leucines. Indeed, typically about 50% of all identical positions are accounted for by leucines in pairwise BAP31 vDED:DED alignments extracted from the multiple alignment shown in Fig. 1c. BAP31 functions in quality control and sorting of a number of client membrane proteins including immunoglobulin D (IgD) [1], major histocompatibility (MHC) class I molecules [5], [6], cystic fibrosis transmembrane regulator (CFTR) [7], beta-2 integrin [8], cellubrevin [9], several members of the MARCH family of membrane associated ubiquitin ligases [10] and the tetraspanins CD9 and CD81 [11]. These proteins are likely recognized and bound as they enter the ER, as BAP31 interacts with components of the Sec translocon including Sec61β and TRAM, and has been shown to bind directly to translocon-locked CFTR [12]. BAP31 controls the fates of its bound clients, which may be either retained in the ER, progress through the secretory pathway or be extruded and subjected to ER-associated degradation[9]–[15]. BAP29 is less well studied, but is known to assist or regulate the function of BAP31 in sorting of some clients including IgD [15] and MHC class I molecules [6]. Most client membrane proteins of BAP31 are probably recognized by the transmembrane domain [2], [5], [9], [10], while the C-terminal cytoplasmic region appears to rather play a role in forming interactions with the cytoskeleton [16] and possibly in docking to the translocon, as Yet3p, a yeast homologue of BAP31, has been reported to require the cytoplasmic domain for efficient interaction with the Sec translocon [17]. It may also be noted that although the cytoplasmic domain is not required for binding to cellubrevin, it is needed for its proper sorting [9]. BAP31 is furthermore involved in apoptosis. It forms for example a complex with the mitochondrial fission 1 (Fis1) membrane protein, which spans the ER and mitochondria and serves as a platform for activation of caspase-8 [18]. In this context, the vDED domain plays an important role, as deleting the domain renders the complex incapable of recruiting caspase-8 [18]. The vDED domain is flanked by two caspase-8 cleavage sites and is therefore excised upon activation of caspase-8. This converts BAP31 from an apoptotic suppressor to an apoptotic activator [4]. Specifically, it has been shown that the membrane-bound cleavage product, p20 BAP31, is highly pro-apoptotic [4], while the fate and possible functions of the excised vDED domain are unknown. Interestingly, the caspase-8 cleavage sites are not conserved in BAP29 in spite of the overall high sequence similarity. Indeed, there are no indications that BAP29 is involved in apoptosis. To shed light on the structure and function of the vDED domains and full-length C-terminal cytoplasmic regions of BAP29 and BAP31, we have investigated their architecture at the secondary, tertiary and quaternary structure levels using a range of biophysical methods.

## Results

### Domain Organization and Secondary Structure

**Figure 2:**
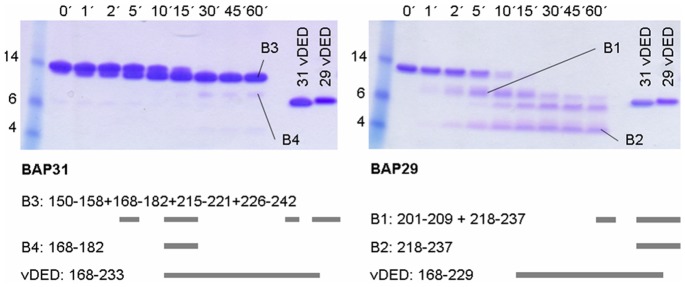
Limited proteolysis of the C-terminal cytoplasmic regions. Samples of the cytoplasmic regions of BAP31 (left) and BAP29 (right) were treated with chymotrypsin and quenched at the indicated time points. For reference, the SDS gels were run together with the untreated vDED domains and a molecular marker (weight for the bands are indicated in kDa). Bands marked B1–B4 were cut out and subjected to in gel trypsination and MALDI TOF mass spectrometry analysis. The detected peptides are listed and depicted graphically beneath the gels and the vDED constructs are shown for reference.

**Figure 3:**
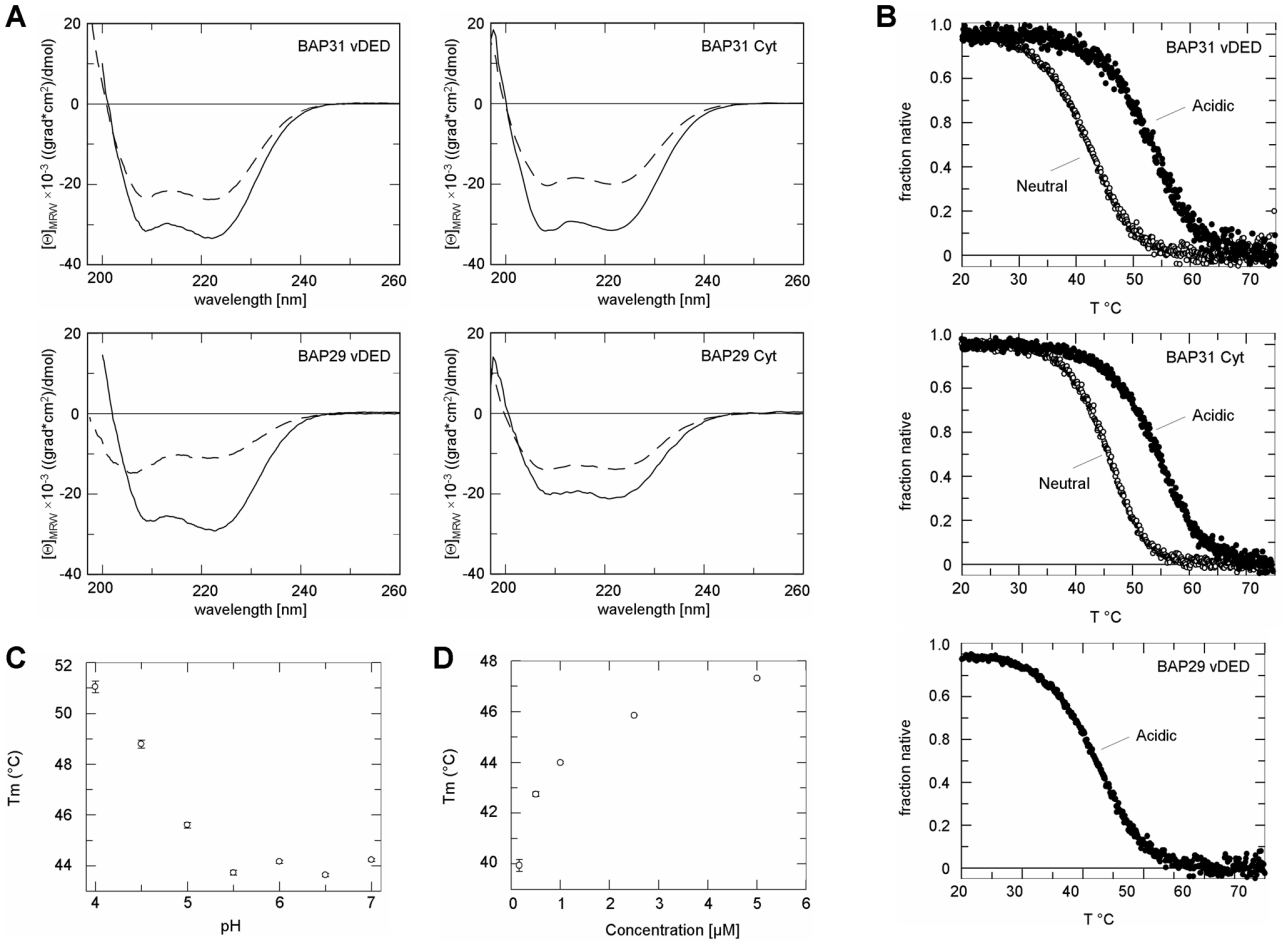
CD spectroscopy of the C-terminal cytoplasmic regions and vDED domains. (**a**) CD spectra of the four expressed BAP29 and BAP31 constructs. Spectra are shown at both pH 7.0 (stippled lines) and pH 4.2 (solid lines) for BAP31 vDED (top left), BAP31 full-length C-terminal cytoplasmic region (top right), BAP29 vDED (bottom left) and BAP29 full-length C-terminal cytoplasmic region (bottom right). (**b**) CD melting curves. Curves are shown for BAP31 vDED (top), BAP31 full-length C-terminal cytoplasmic region (middle) and BAP29 vDED (bottom). For BAP31, curves were obtained at both pH 7.0 and pH 4.2 as annotated on the figure. (**c**) Effect of pH on stability. Plot shows the midpoint of thermal unfolding of the BAP31 C-terminal cytoplasmic region as a function of pH at a protein concentration of 1 µM. (**d**) Effect of protein concentration on stability. Plot shows the midpoint of thermal unfolding of the BAP31 C-terminal cytoplasmic region as a function of protein concentration at pH 6.0.

In order to address the structure and stability of the C-terminal cytoplasmic regions of BAP29 and BAP31 we first employed limited chymotrypsin proteolysis (Fig. 2). The C-terminal region of BAP31 quickly degraded into a slightly smaller species, which was however quite stable towards further degradation. In contrast, the C-terminal region of BAP29 was extensively degraded, indicating that it exists in a much more extended and less well folded conformation than that of BAP31 (Fig. 2). From each protein, two chymotrypsin digestion products were further digested by in gel trypsination and analyzed using mass spectrometry (MS). The four chymotrypsin digestion products were found to all overlap, or partially overlap, with the vDED domain (Fig. 2). Next we analyzed all four constructs using CD spectroscopy (Fig. 3, Table 1). Surprisingly, we found that the spectra were markedly different at neutral and acidic pH i.e. pH 7.0 and pH 4.2 (Fig. 3a). Secondary structure analysis of the deconvoluted spectra suggests a high α-helical content for BAP31 with values of 79% at pH 7.0 and 100% at pH 4.2 (Table 1). The length of the vDED domain and full-length C-terminal cytoplasmic region of BAP31 are 67 and 120 residues respectively including the two additional N-terminal residues left after tag cleavage. Thus, if the vDED domain is the only α-helical entity in the C-terminal cytoplasmic region, the α-helical contents of the latter should be 79% × (67/120) = 44% at pH 7.0 and 100% × (67/120) = 56% at pH 4.2, yet the measured values are much higher; 63% and 98% respectively (Table 1). We therefore conclude that additional α-helical structure is present in the C-terminal cytoplasmic region, which is also in good agreement with the limited proteolysis experiment. The presence of coiled coils can be evaluated from CD spectra. The [Θ]_222_/[Θ]_208_ ratio is thus generally in the range of 0.8–0.9 for non-associated helices and 1.0–1.1 for dimeric coiled coils[19]–[21]. For BAP31 vDED we obtained values >1.0 regardless of the pH, and for the full-length C-terminal region, the values were just slightly under 1.0 (Table 1). This strongly suggests that the vDED domain forms a coiled coil over a wide pH range and indicates that the additional structure present in the C-terminal region may also consist partly or mainly of coiled coil. In the case of BAP29, it is clear from visual inspection of the spectra that the vDED domain contains little α-helix at pH 7.0 while a strong increase in α-helical structure is induced at pH 4.2 (Fig. 3a). Calculated α-helical contents and [Θ]_222_/[Θ]_208_ values are 28% and 0.79 at pH 7.0 and 88% and 1.12 at pH 4.2 (Table 1). We therefore conclude that BAP29 vDED is partially disordered at pH 7.0 but very likely adopts a coiled coil fold at pH 4.2. For the full-length C-terminal cytoplasmic region of BAP29, the measured α-helical content is 35% at pH 7.0 and 58% at pH 4.2. The C-terminal cytoplasmic region is thus rather poorly structured at pH 7.0, as also indicated by the limited proteolysis results, whereas structure is acquired at pH 4.2, as also observed for the vDED domain alone. However the structural content at pH 4.2 is not very different from the 50% expected if the vDED domain is the only α-helical entity in the domain (using the same type of calculation as described above for the C-terminal cytoplasmic region of BAP31). Thus, considering the rather low accuracy associated with calculating secondary structure content from CD spectra, we can neither confirm nor rule out that additional structure is present in the cytoplasmic region of BAP29 at pH 4.2.

**Table 1:** CD results.

Protein	pH	Helix (%)	β-strand (%)	Random coil (%)	[Θ]_222_/[Θ]_208_	T_m_ (°C)
BAP31 vDED	7.0	79	0	21	1.02	42.12± 0.12
	4.2	100	0	0	1.08	53.12± 0.17
BAP31 Cyt	7.0	63	6	31	0.98	45.72± 0.05
	4.2	98	0	2	0.99	54.41± 0.11
BAP29 vDED	7.0	28	16	56	0.79	< 20
	4.2	88	0	12	1.12	42.77 ± 0.17
BAP29 Cyt	7.0	35	15	50	0.97	n.d.
	4.2	58	8	34	1.05	n.d.

Overview table showing calculated secondary structure content, [Θ]_222_/[Θ]_208_ ratio and melting temperature for all tested constructs at both pH 7.0 and pH 4.2.

### Thermal Stability

Next we measured the stability of the expressed constructs against heat denaturation using CD spectroscopy (Fig. 3b). BAP31 vDED exhibits a rather modest midpoint of thermal unfolding (apparent melting temperature) of 42.1 °C at pH 7.0 (Table 1). For the full-length C-terminal region, the melting temperature is 3.6 °C higher (Table 1), which is congruent with the notion that a second domain or sub-domain is present here. Both constructs furthermore showed a highly increased stability at pH 4.2 (shift in melting temperature of 8.7–11 °C) (Fig. 3b, Table 1). BAP29 vDED is much less stable than BAP31 vDED. No cooperative melting curve could be obtained at pH 7.0 due to the domain being poorly structured at this pH, and at pH 4.2 the melting temperature is 10.4 °C lower than for BAP31 vDED (Fig. 3b, Table 1). Next we investigated the thermal stability of the full-length C-terminal region of BAP31 at a range of pH values. Here we found that the stability against heat denaturation is approximately unchanged within a pH range of 5.5–7, but starts to increase when the pH is lowered to 5 and increases further at 4.5 and again at 4 (Fig. 3c). Finally, we also studied the effect of protein concentration on stability of the full-length C-terminal cytoplasmic region of BAP31. Here we found an increase in stability with increased concentration, indicative of oligomeric assembly (Fig. 3d). This result is thus in good agreement with the notion that one or two coiled coils are present in BAP31.

### Three Dimensional Structure of the vDED Domain of BAP31

**Figure 4:**
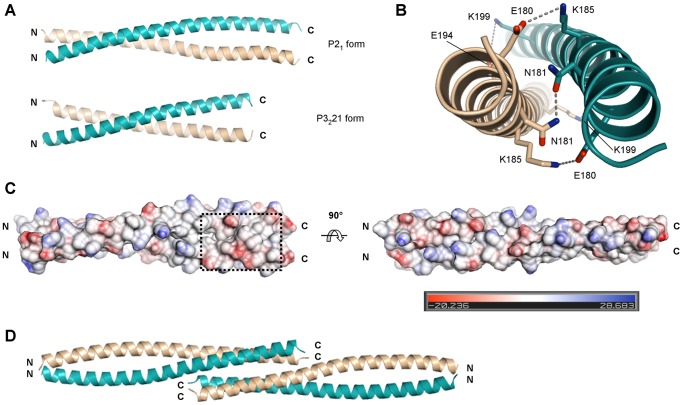
Structures of the BAP31 vDED domain. (**a**) Structural overview of the two crystal forms. (**b**) Selected inter-helical interactions. Side chain interactions discussed in the text are shown for the P2_1_ form. Stippled lines indicate hydrogen bonds or ionic interactions. (**c**) Charge distribution at the surface. The P2_1_ form is colored by electrostatic potential (inset shows color code) and shown in two orientations; the orientation on the left is the same as in the top panel in (a). The stippled box denotes a small predominantly acidic region. (**d**) Putative tetramer. In the P2_1_ form, two dimers form a tail-to-tail tetramer across a crystal contact, which is predicted by the PISA server to be stable in solution.

The structure of the vDED domain of BAP31 was determined in two different crystal forms; P2_1_ at pH 8.0 and twinned P3_2_21 at pH 4.2 (Fig. 4a). The vDED domain forms an uninterrupted parallel coiled coil dimer in both structures. Almost all residues could be modeled in the P2_1_ form (172–233), but not in the P3_2_21 form (169–220). Apart from that, the two structures are however quite similar and exhibit a RMSD Cα value of 1.12 Å for 94 aligned residues. It is surprising that part of the structure was disordered at pH 4.2, as the CD experiments show that the domain is considerably more stable at this pH than at neutral pH. We have no obvious explanation for this observation, but one possibility is that certain buffer molecules in the crystallization condition e.g. LiSO_4_ lead to partial unwinding of the coiled coil. In coiled coils, a series of consecutive heptad sequence repeats can generally be recognized where the first and fourth positions, i.e. the ‘a’ and ‘d’ positions, are predominantly hydrophobic. This is also the case for the vDED domains of BAP29 and BAP31 (Fig. 1b). In dimeric coiled coils, the residues in these positions form the center of the dimer interface i.e. the hydrophobic core. Most ‘a’ and ‘d’ positions are indeed hydrophobic in BAP31 vDED, but interestingly the N-terminal most ‘a’ position is an asparagine, Asn-181 (Fig. 4b). Another typical feature of dimeric coiled coils is the presence of inter-helical salt bridges between ‘e’ and ‘g’ positions, most typically formed by lysines and glutamates [22]. There are at least two such salt bridges in BAP31 vDED; Glu-180:Lys-185 and Glu-194:Lys-199 (Fig. 4b). In addition there is also a clear potential for a salt bridge between Lys-201 and Glu-206, but in both crystal forms, these residues form crystal contacts instead. Notably, none of these 2–3 salt bridges are conserved in BAP29. Furthermore, Leu-188, which occupies one of the ‘a’ positions in BAP31 is a glutamine in BAP29 (Gln-184). This is much less favorable and BAP29 would therefore be expected to form a less stable coiled coil than BAP31, which is in good agreement with the CD analysis. All other ‘a’ and ‘d’ positions are fully conserved between the two proteins. The vDED domains of both BAP29 and BAP31 contain many charged residues. These charges are however more or less evenly spread over the surface of BAP31 vDED rather than being organized in extended acidic or basic regions, except that a small predominantly acidic patch is found in the C-terminal end (Fig. 4c).

### Quaternary Structure and Heteromeric Interactions

**Figure 5:**
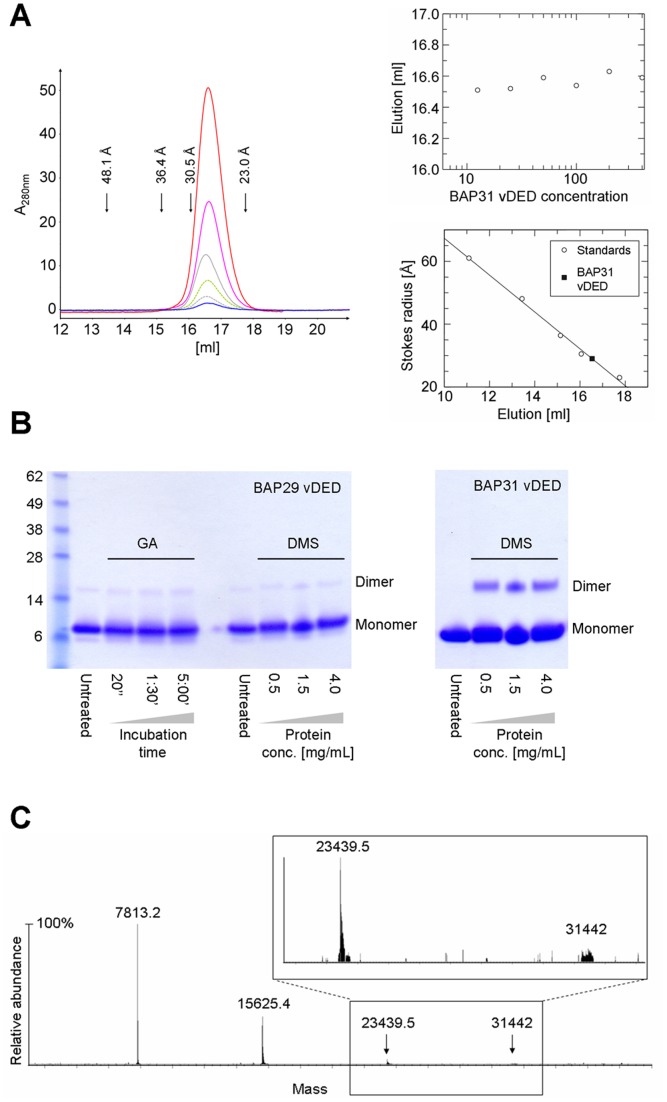
Quaternary structure of the vDED domain. (**a**) Gel filtration analysis of BAP31 vDED at pH 7.0. Different concentrations of BAP31 vDED were used; 10 µM (blue), 25 µM (grey), 50 µM (green), 100 µM (grey), 200 µM (pink) and 400 µM (red). As seen in the top right graph, the elution volume was approximately the same for all concentrations (average is 16.54 mL). The column was calibrated with aldolase (Stoke’s radius of 48.1 Å), conalbumin (36.4 Å), ovalbumin (30.5 Å), and carbonic anhydrase (23 Å). Plotting elution volume against Stoke’s radius of these standards (bottom right) allowed us to estimate the Stokes’ radius of BAP31 vDED to 28.97. (**b**) Chemical cross-linking analysis of BAP29 vDED and BAP31 vDED. BAP29 vDED was cross-linked with glutaraldehyde (labeled GA on the figure) at pH 7.5 and the reaction was quenched at different time points as indicated beneath the gel. The weak dimer band seen is due to spontaneous disulfide bridge formation (it is also present in the untreated sample). Cross-linking of BAP31 vDED with glutaraldehyde resulted in aggregation (not shown). Both BAP29 vDED and BAP31 vDED were also cross-linked with DMS at pH 7.5 at various protein concentrations as indicated in the figure. (**c**) Native ESI mass spectrometry analysis of BAP31 vDED. A substantial peak was seen for the dimer species (≥35% of the monomer peak height) while peaks for the trimeric and tetrameric species were minuscule (∼3% and <1% respectively of the monomer height).

**Figure 6:**
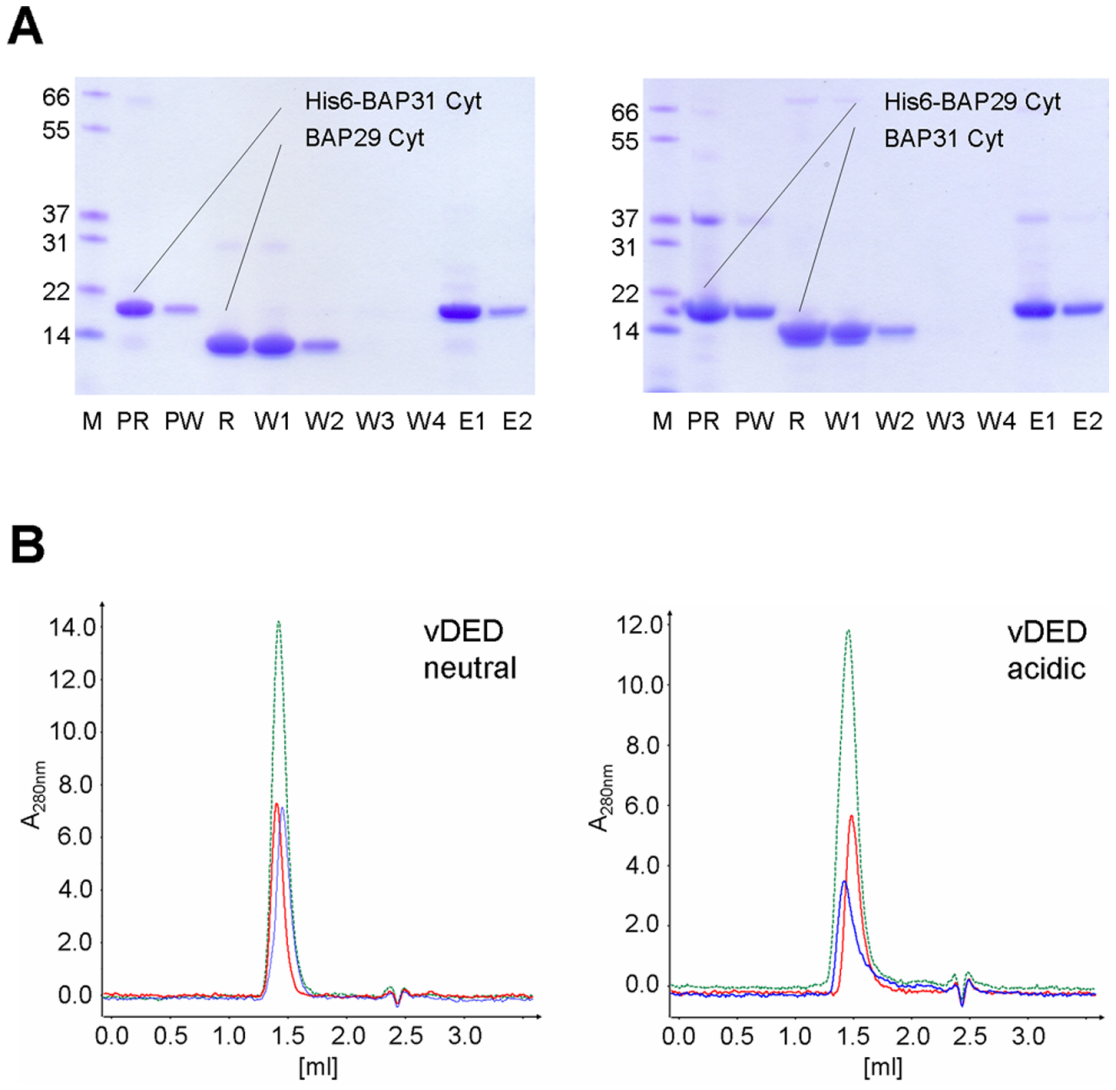
BAP29–BAP31 interaction studies. (**a**) IMAC pull-down. Left; His-tagged full-length C-terminal cytoplasmic domain of BAP31 was adsorbed to the nickel resin (run fraction; lane PR) and excess protein was washed off (PW). Purified untagged BAP29 full-length cytoplasmic domain was then applied to the column (run fraction; lane R). The column was then thoroughly washed (W1–W4) and eluted with high concentrations of imidazole (E1, E2). No co-elution of BAP29 was observed. Right, as for the left panel, but BAP29 was adsorbed and BAP31 applied to the column afterwards. The dimer bands seen on the gels for BAP29 are due to spontaneous disulfide bridge formation. (**b**) Analytical gel filtration. Runs were conducted at both pH 7.0 (left) and pH 4.2 (right) with BAP31 vDED alone (red), BAP29 vDED alone (blue) or both proteins together (green). No shifts were observed, indicating that a complex is not formed. Similar gel filtration runs were also conducted for the full-length C-terminal cytoplasmic domains and combinations of the vDED and full-length domains. Also here, no complex formation was observed (results not shown).

In the P2_1_ form of BAP31 vDED, residues 204–233 of two dimers interact to form a tail-to-tail four-helical bundle across a crystal contact (Fig. 4d). This dimer-dimer interface covers an area of 2900 Å^2^ and is predicted by the PISA server to be stable in solution. To determine the oligomeric state in solution we first employed calibrated gel filtration at pH 7 (Fig. 5a). Here the Stoke’s radius was found to be 28.97 Å. For comparison, the radii of gyration (related to and numerically similar to the Stoke’s radius) of the dimer and potential tetramer of BAP31 vDED were estimated to be 29 and 39 Å respectively based on the P2_1_ structure. We therefore conclude that BAP31 vDED elute as a dimer on the gel filtration column at pH 7.0. Next, we employed chemical cross-linking at pH 7.5, which suggested that BAP31 vDED forms either a dimer or a mixture of monomers or dimers. No evidence was found for the formation of tetramers. Finally, we also investigated the oligomeric state of BAP31 vDED using native electrospray ionization (ESI) mass spectrometry at pH 7.0. Here we found clear evidence for the presence of dimers, while only a very negligible signal was observed for the tetrameric species (Fig. 5c). We therefore conclude that the observed tetramerization interface is unlikely to be physiologically relevant. The quaternary structure of BAP29 vDED was also investigated, though only by chemical cross-linking. Here the results indicated a monomeric configuration at pH 7.5 (Fig. 5b). This is in good agreement with the limited proteolysis and CD results, which showed that this domain adopts a poorly structured conformation at pH 7.0. It was also tested if the cytoplasmic regions of BAP29 and BAP31 are capable of forming a heteromeric complex. However, neither IMAC pull-down nor analytical gel filtration supported this notion (Fig. 6). Furthermore, an attempt to form a heterodimeric complex by denaturing and renaturing the vDED domains together was unsuccessful (Fig. S1).

## Discussion

### The vDED Domain of BAP31 Forms a Dimeric Coiled Coil

The vDED domain of BAP31 is predicted to form a coiled coil, but also shares weak sequence similarity with DED domains (10–18% for the DED domains analyzed here; Fig. 1c), which has been the source of some confusion regarding its structure and function. The structures presented here now show for the first time that the vDED domain adopts a dimeric coiled coil fold with no structural similarity to DED domains. About half of the sequence similarity between BAP31 vDED and the DED domains used in our sequence analysis can be accounted for by leucines. Based on this, we propose that the apparent weak homology between vDED and DED domains is an artifact caused in part by the fact that DED domains have a strikingly high content of leucine (average of 20% for the DED domains analyzed here) in likeness with most coiled coils -including BAP31 vDED (leucine content of 14%). In addition, it may be expected that both types of domains also have a high content of other residues with high propensities for forming α-helix, which may result in additional coincidental identities. If this hypothesis is valid, it would be expected that DED domains also share weak sequence similarities with other coiled coil domains. Indeed, it has been recently shown that the so called ‘pseudo DED’ (pDED) domain of HIP1 adopts a dimeric coiled coil fold [23], and also other DED-like domains have been predicted to form coiled coils [24]. It is thus clear that weak sequence similarity to DED domains should be interpreted with great caution. The vDED domains of BAP29 and BAP31 contain many charged residues, as was already noted when they were originally cloned [1]. This is however not unusual for dimeric coiled coil domains, as hydrophobic residues are disfavored in all but the ‘a’ and ‘d’ positions of the heptad sequence repeats [25]. We find that these charged residues are largely evenly spread over the surface of the domain except for a small, possibly functionally relevant, C-terminal patch of predominantly acidic residues. It is noteworthy that no extensive basic regions could be identified, as this strongly suggests that the domain does not form appreciable interactions with the membrane surface. Using analytical gel filtration, chemical cross-linking and native ESI mass spectrometry, we have shown that BAP31 vDED also forms a dimer in solution or a mixture of monomers and dimers. We therefore conclude that BAP31 probably functions as a dimer *in vivo*, or alternatively as a multimer assembled from two or more dimers interacting with each other *via* the N-terminal membrane-bound domain. It is noteworthy that a conserved asparagine, Asn-181, is found in one of the ‘a’ positions in the hydrophobic core /dimer interface. Asparagines in ‘a’ positions strongly favor dimer formation over trimers and tetramers and are often important determinants of strand polarity and axial register [26], [27]. In addition they have also been shown to markedly lower dimer stability[26]–[28], in particular when they, as Asn-181, are found N-terminally in the domain [28]. Indeed, using CD spectroscopy, we found that BAP31 vDED has a fairly modest melting temperature of 42.1 °C under near physiological conditions. This may be highly relevant in enabling dynamic assembly and interactions of the domain *in vivo.*

### The C-terminal Cytoplasmic Region of BAP31 Likely Contains a Second Coiled Coil

Limited proteolysis revealed that the C-terminal cytoplasmic region of BAP31 contains a stable core that is considerably longer than the vDED domain and only slightly smaller than the full-length cytoplasmic region. Furthermore, CD measurements revealed that it contains a higher α-helical content than can be accounted for by the vDED domain alone and exhibits a 3.6 °C higher midpoint of thermal unfolding under identical (near physiological) conditions. Taken together, this suggests that an additional α-helical domain or sub-domain is present. Alternatively, it could be speculated that the vDED domain is not a domain in itself, but an integrated part of a single coiled coil encompassing most of the C-terminal cytoplasmic region. This is however highly unlikely, as the vDED domain is flanked on either side by a functional cleavage site for caspase-8 [4], which strongly indicates the presence of flexible loops. As a second coiled coil is predicted in the C-terminal region (Fig. 1a), and as the measured [Θ]_222_/[Θ]_208_ ratio is close to the value expected for coiled coils, we conclude that the additional α-helical structure in the cytoplasmic region likely represents a second smaller coiled coil domain. It is however uncertain if the same is true for BAP29. The C-terminal cytoplasmic region of this protein is partially unfolded at pH 7.0, and although structure is acquired at pH 4.2 (see below) there is no clear indication that it extends beyond the vDED domain.

### The C-terminal Cytoplasmic Regions and vDED Domains are Highly pH Sensitive

A surprising outcome from this study is the finding that the cytoplasmic domains are highly pH sensitive. In the case of BAP29, limited proteolysis and chemical cross-linking results showed that the full-length cytoplasmic region adopts a proteolytically unstable and most likely monomeric structure at pH 7.0. CD measurements furthermore revealed that both the vDED domain and the full-length C-terminal cytoplasmic region contain no coiled coils and overall little secondary structure at pH 7.0. However, at pH 4.2, the vDED domain exhibited a typical coiled coil CD spectrum. We therefore conclude that the cytoplasmic region of BAP29 is partially unfolded at pH 7.0 but adopts a coiled coil fold at pH 4.2. The reason why a coiled coil is not formed at pH 7.0 is not clear, but one possibility is that the N-terminal of BAP29 vDED, which is highly acidic, would cause charge repulsion. In contrast, BAP31 vDED forms a coiled coil over a wide pH spectrum although the melting temperature is 11 °C higher at pH 4.2 than at pH 7.0. This dramatic increase in stability is puzzling since there is no clear evidence for any substantial charge repulsion in the dimer interface at pH 7.0 (though Glu-174 may perhaps cause some level of repulsion), and since very low pH may in fact destabilize the 2–3 inter-helical salt bridges that are present in the domain through protonation of acidic residues. However, several cases have been reported of coiled coils being stabilized at highly acidic pH in spite of a loss of salt bridges [29]. A possible explanation for this is that the loss of ionic interactions at acidic pH may be counteracted by favorable interactions between basic residues and anions present in the solvent, reminiscent of the counterion condensation observed in double stranded DNA [30]. The melting temperature of BAP31 vDED is 10.4 °C higher than for BAP29 vDED at pH 4.2. It is thus clear that BAP29 vDED is generally less well disposed for forming a stable coiled coil dimer than BAP31 vDED. This is however also what would be expected when considering the sequence of BAP29 vDED in context of the BAP31 vDED structure. Thus, a leucine in one of the ‘a’ positions in the hydrophobic core /dimer interface of BAP31 is substituted by a less favorable glutamine in BAP29 and none of the two or three inter-helical salt bridges found in BAP31 are conserved. This lower coiled coil propensity may also be part of the reason why BAP29 vDED only forms a coiled coil under acidic conditions. Additional favorable interactions induced at pH 4.2, i.e. hydrogen bonds between acidic residues and /or favorable interactions with the surrounding salt, may thus be required to sufficiently stabilize the fold. The pH of the cytoplasm is normally near neutral and it is therefore unclear if the observed effects of pH are of any physiological significance. Acidification does happen during apoptosis, but this drop in pH is usually measured to be in the range of 0.3–0.4 units [31]. It is therefore unlikely that a pH as low as 4–5 is ever experienced by BAP29 or BAP31 in the cell. On the other hand, we also find it somewhat unlikely that the coiled coil potential of BAP29 is never realized *in vivo*. We therefore hypothesize that folding and assembly of BAP29 vDED *in vivo* may be governed mainly or entirely by other parameters than pH. Such parameters could for example be post-translational processing, binding of specific ions or other solutes, interaction with cognate protein partners etc. It may also be mentioned that a case very similar to that of BAP29 vDED has been reported for another human coiled coil domain; the leucine zipper of Par-4. This domain thus also forms a partially unfolded monomer at pH 7.0 but a coiled coil dimer at acidic pH [32]. New insights into what governs the folding of this domain *in vivo* may thus also be of relevance to BAP29 and *vice versa*.

### The Cytoplasmic Domains of BAP29 and BAP31 may not Interact Directly

BAP29 and BAP31 were originally discovered as interaction partners for IgD [1], and as they bind simultaneously to this protein in roughly equimolar amounts, it seems likely that they are capable of forming a heteromeric complex [1], [15]. This notion has also recently been supported by FRET experiments [33]. It has furthermore been reported that BAP29 and BAP31 can be shown biochemically to form heterodimers [2], although it is unclear which experiments were used to arrive at this conclusion (results were not shown). Using IMAC pull-down and analytical gel filtration, we found no evidence for direct interaction of the cytoplasmic domains of BAP29 and BAP31 at neither pH 4.2 nor pH 7.0, and we therefore suggest that BAP29 and BAP31 either interact *via* mediator proteins or perhaps more likely *via* their transmembrane domains. Alternatively it is possible that nascent BAP29 and BAP31 molecules can form not only homodimeric but also heterodimeric vDED coiled coils, as has been suggested previously [2]. However, when we denatured and renatured the two vDED domains together, no heterodimeric complex was formed. Indeed, the fact that a leucine in an ‘a’ position in BAP31 (Leu-188) is substituted by a glutamine in BAP29 (Gln-184) is very likely to prevent heterodimerization, or at least heterodimerization in axial register.

## Materials and Methods

### Reagents

Terrific broth (TB) was from Formedium, isopropyl β-D-1-thiogalactopyranoside (IPTG) was from Saveen Werner, nickel nitrilotriacetic acid (Ni-NTA) agarose beads were from Life Technologies, glutaraldehyde was from Fluka, dimethyl suberimidate (DMS) was from Thermo Scientific, SDS-PAGE gels were from Life Technologies and crystallization reagents were from Qiagen. All other chemicals were from Sigma-Aldrich.

### Protein Expression and Purification

Genes coding for the full-length C-terminal cytoplasmic region of human BAP29 (UniProt: Q9UHQ4, residues 125–237) and BAP31 (UniProt: P51572, 124–242) as well as genes coding for the vDED domains of BAP29 (168–229) and BAP31 (168–233) were amplified from cDNA obtained from the human ORFeome library [34] and cloned into the pNIC28-Bsa4 vector [35] using ligation independent cloning. This vector adds an N-terminal hexa-histidine tag followed by a tobacco etch virus (TEV) cleavage site to the over-expressed protein. The theoretical pI of the expressed constructs are 5.19 (BAP31 vDED), 5.06 (BAP31 full-length cytoplasmic), 4.73 (BAP29 vDED) and 6.73 (BAP29 full-length cytoplasmic). The four constructs were transformed into *E. coli* BL21(DE3) Rosetta^TM^ 2 expression cells (Novagen). For production of native unlabeled protein, cells were grown in TB medium and induced with 0.2 mM IPTG at 20 °C (over night incubation). After cell harvesting and lysis, the protein was purified by IMAC chromatography using either an open gravity flow column (Biorad) loaded with Ni-NTA agarose beads or an ÄKTAexplorer^TM^ equipped with a 5 mL HisTrap^TM^ FF fast flow column (GE Healthcare). All buffers contained 0–2 mM Tris(2-carboxyethyl)phosphine (TCEP); high concentrations of TCEP was used for BAP29 as it is prone to form inter-molecular disulfide bridges, while it was generally left out entirely for the BAP31 constructs, as they contain no cysteines. After thorough washing, the bound protein was eluted with high concentrations of imidazole and cleaved over night with ∼1 mg recombinant TEV protease per 20 mg eluted protein, while dialyzing against gel filtration buffer (20 mM Tris-HCl pH 8, 150 mM NaCl and 0–2 mM TCEP). The cleaved protein was then passed over the IMAC column again to separate it from uncleaved protein and TEV protease (reverse IMAC purification) and further purified by gel filtration on an ÄKTAexplorer^TM^ equipped with a Superdex 75 or 200 column (GE Healthcare). The purified protein was concentrated using a Vivaspin 20 concentrator (Sartorius) with 10 kDa cut-off. For production of selenomethionine labeled BAP31 vDED, cells were grown in minimal medium according to standard protocol (adapted from Van Duyne et. al. [36]) and purification was carried out as for the native protein, except that all buffers contained 2 mM TCEP to keep the selenomethionine reduced.

### Limited Proteolysis

Stability tests for the expressed BAP constructs against proteolytic degradation was performed at neutral pH at room temperature on purified protein in the presence of chymotrypsin (chymotrypsin:target protein ratio: 1∶1000). The reaction was stopped after different time points by addition of SDS sample buffer and subsequent heating. The samples were then analyzed on SDS-PAGE. Selected bands were excised from the gel, and the derived peptides were analyzed using MALDI TOF mass spectrometry.

### CD Spectroscopy

CD spectra of the expressed BAP constructs were recorded with a JASCO J600A spectropolarimeter (0.1 cm cell length, 2–10 µM protein concentration, 1 nm bandwidth) and corrected for buffer contributions (acidic CD buffer; 20 mM Na-citrate pH 4.2, 150 mM NaCl, 0.1 mM TCEP, neutral CD buffer; 20 mM Na-P pH 7.0, 150 mM NaCl, 0.1 mM TCEP). Corrected CD spectra were analyzed with the online tool Dichroweb [37], [38] using the K2D algorithm [39]. Temperature-induced unfolding transitions were monitored via CD spectroscopy at 228 nm from 20–80 °C. Experimental data were analyzed according to a 2-state model using GraFit 5 (Erithacus Software Ltd.). Protein concentration of the various BAP constructs was 1 µM in the same buffers as stated above (for the pH dependence, a buffer mixture containing Na-citrate and Na-P was used). To study the influence of concentration on stability of the BAP31 C-terminal cytoplasmic region, unfolding curves were recorded at pH 6.0 with concentrations from 0.25 µM to 5 µM.

### Determination of Quaternary Structure

To assess the oligomeric structure of BAP31 vDED, protein samples in the concentration range from 10–400 µM were analyzed on a 24 ml gel filtration column (Superdex 200 5/150 GL, GE Healthcare) in the neutral CD buffer (see above). The column was calibrated with a number of reference proteins under identical conditions and the result was compared to the radius of gyration of the BAP31 vDED dimer and potential tetramer as estimated from the P2_1_ structure using HYDROPRO 10 (in mode 1) [40]. For the glutaraldehyde and DMS chemical cross-linking experiments, the protein was first dialyzed against 20 mM HEPES pH 7.5 and 150 mM NaCl. For BAP29, we also added 20 mM TCEP to the dialysis buffer in order to reduce intermolecular disulfide bridges as much as possible. For the glutaraldehyde experiments, we added 0.024% glutaraldehyde to ∼0.5 mg/mL protein at room temperature and quenched the reactions at various time points with ∼90 mM Tris-HCl pH 8.0. For the DMS experiments we added 5.5 mM DMS to protein in concentrations of 0.5, 1.5 and 4.0 mg/mL corresponding to a molar ratio of DMS:protein of ∼10–85. The reactions were then incubated for 1 hour at room temperature and quenched with Tris-HCl. The cross-linked samples were then analyzed on SDS-PAGE. For native ESI MS, purified BAP31 vDED was dialyzed against 150 mM NH_4_ acetate pH 7 and diluted to 1.24 mg/mL. The sample was further diluted in 15% acetonitrile and 5% 2-propanol and a few µL of that were directly injected into a LTQ-Orbitrap Velos mass spectrometer (Thermo Scientific) using a TriVersa Nanomate (Advion Biosciences) interface. MS data were acquired over a mass range of m/z 500 to m/z 3000 at a resolution of 100’000 at m/z 400. Most prominent charge states of the monomer and dimer were detected between z = 5 to z = 8. Deconvolution of the data was performed using a non-commercial software.

### Interaction Studies

For the pull-down experiments, we used open gravity flow columns with 50 µL Ni-NTA beads and buffers consisting of 20 mM Na-P pH 7, 150 mM NaCl, 0.5 mM TCEP and 30 mM imidazole (wash buffer) or 500 mM imidazole (elution buffer). The experiments were carried out at 4 °C. The column was first over-loaded with His-tagged protein (full-length C-terminal cytoplasmic domain of BAP29 or BAP31) and the excess was then washed off with wash buffer (10 column volumes). Then an excess of the untagged putative interactor was run over the column followed by four washing steps (5 column volumes each). Elution was then done with elution buffer in two steps (5 column volumes each). All samples were then analyzed on SDS-PAGE. Interactions between the expressed BAP29 and BAP31 constructs were also probed using an analytical gel filtration setup; the proteins alone or the potential complex (at concentrations of 75 µM) were applied to a Superdex^TM^ 200 5/150 GL analytical gel filtration column using an ÄKTAmicro^TM^ chromatography system (GE Healthcare) equipped with an A-905 Autosampler, which automatically injected 25 µl of protein sample. Analytical gel filtration runs were performed at 4 °C at a flow rate of 0.2 ml/min in the neutral CD buffer.

### Crystallization and Data Collection

Two crystal forms were obtained for the vDED domain of BAP31; P2_1_ and P3_2_21. They were both obtained by vapor diffusion in drops consisting of 2 µL protein and 2 µL of the crystallization condition. Selenomethionine labeled crystals of the P3_2_21 form of BAP31 vDED were obtained at 19 °C using protein at a concentration of 14.1 mg/mL. The crystal used for data collection was roughly 350×180×180 µm and grew in a condition containing 100 mM Na citrate pH 4.2, 33% PEG 400 and 200 mM LiSO_4_. This crystal was flash frozen in liquid nitrogen without prior soaking. Crystals of the P2_1_ form of BAP31 vDED were obtained at 4 °C using native unlabeled protein that had been dialyzed into 150 mM NH_4_ acetate pH 7 and concentrated to 12.4 mg/mL. The crystal used for data collection was about 250×40×20 µm and was grown in 100 mM Tris-HCl pH 8.0, 34% PEG 2000 monomethyl ether and 150 mM KBr. The crystal was lifted into a drop containing 100 mM Tris-HCl pH 8.0, 33% PEG2000 monomethyl ether, 150 mM KBr and 20% glycerol and soaked for 30′′–3′ before it was frozen. Both data sets were collected at the Diamond light source synchrotron (DLS) in England at beamline I02.

### Structure Determination

Both data sets were processed using XDS and XSCALE [41] (Table 2). The crystallization conditions, crystal morphology and cell dimensions for the P3_2_21 form are essentially the same as for a previously described crystal form of BAP31 vDED, which was reported to belong to space group P6_1_22 or P6_5_22 [42]. Indeed, the P3_2_21 form could also be scaled in the P622 point group with good statistics, but it was clear from the twinning diagnostics reported by Phenix Xtriage [43] that the data are twinned (Fig. S2 shows results of the L-test). As twinning is not possible in the P622 point group, we concluded that this crystal form belongs to a twinned lower symmetry point group, most likely P6, P312 or P321. Using the anomalous signal to find the selenium sites with HKL2MAP [44] we concluded that the correct space group is P3_2_21. The twin law is merohedral –h, –k, l (only twin law possible in this space group) and the twin fraction was estimated to be 0.436 (maximum likelihood), 0.440 (Britton analysis) or 0.447 (H-test). The apparent P6_5_22 symmetry of the crystal is caused by the presence of a two-fold twin axis parallel to the crystallographic trigonal screw axis. Selenomethionine SAD phasing and auto building was conveniently carried out using the Auto-Rickshaw pipeline [45]. The main steps here were SAD phasing using Phaser [46], density modification using PIRATE [47], and autobuilding using ARP/wARP [48]. We then corrected and refined the resulting model by several cycles of manual rebuilding in Coot [49] and restrained refinement with REFMAC5 in twin mode [50]. We used Phenix with the “use lattice symmetry” option to create an unbiased R_free_ set. The twin fraction was found to be 0.457. When, as a test, the last round of refinement was repeated without taking twinning into account, R_work_ and R_free_ increased greatly to 0.280 and 0.313 respectively. For the P2_1_ form, phasing was achieved by molecular replacement in Phaser using a partially build model of the P3_2_21 form as search model. Auto-Rickshaw then performed several cycles of density modification and auto-building using PIRATE and ARP/wARP. The resulting model was then finally corrected and refined by several cycles of manual rebuilding in Coot and refinement with PHENIX refine [43] using ‘translation libration screw’ (TLS) restraints. Refinement statistics are shown in Table 2. MolProbity v4.01 [51] was used to validate that the models have sound geometry; all residues were in favored regions of the Ramachandran plots and the clash scores /overall scores were 3.52 (99^th^ percentile) /1.14 (100^th^ percentile) for the P3_2_21 form and 0.73 (100^th^ percentile) /0.73 (100^th^ percentile) for the P2_1_ form.

**Table 2:** Data processing and refinement statistics.

	BAP31 vDEDacidic pHPDB: 4JZP	BAP31 vDEDalkaline pHPDB: 4JZL
**Data collection**		
Beamline	DLS I02	DLS I02
Wavelength (Å)	0.9780	1.0000
Space group	P3_2_21	P2_1_
Cell dimensions		
*a*, *b*, *c* (Å)	70.61, 70.61, 80.12	56.69, 42.90, 62.67
*α, β, γ* (°)	90, 90, 120	90, 115.87, 90
Resolution (Å)	28.6–2.1 (2.21–2.10)	28.3–2.2 (2.26–2.20)
*R*_sym_ (%)	4.6 (42.4)	6.7 (57.5)
*I* / *σI*	24.28 (4.0)	15.82 (3.10)
Completeness (%)	99.5 (99.7)	98.5 (98.3)
Redundancy	6.2 (6.2)	5.2 (5.1)
Wilson *B*-factor (Å^2^)	40.3	32.6
**Refinement**		
No. reflections	13261	13771
No. mol /ASU	2	4
*R*_work_ / *R*_free_ (%)	16.2 /18.0	22.0/24.6
Twin law	merohedral –h, –k, l	–
Twin fraction	0.457	–
No. atoms		
Protein	801	2004
Solvent (water)	74	114
Solvent (other)	31	6
*B*-factors		
Protein	49.7	43.7
Solvent	65.9	46.4
R.m.s. deviations		
Bond lengths (Å)	0.006	0.004
Bond angles (°)	0.959	0.680
Ramachandran plot		
Favored (%)	100	100
Outliers (%)	0	0

Numbers in parentheses indicate statistics for the outer shell.

### Sequence and Structural Analysis

All servers were used with default settings. The Coils http://embnet.vital-it.ch/software/COILS_form.html [52] and Paircoil2 http://groups.csail.mit.edu/cb/paircoil2/paircoil2.html [53] servers were used for coiled coil prediction. MEMSAT-SVM http://bioinf.cs.ucl.ac.uk/psipred/ [54] was used for transmembrane helix prediction. Pairwise and multiple sequence alignments were generated using EMBOSS needle http://www.ebi.ac.uk/Tools/psa/emboss_needle/ [55] and Clustal W2 http://www.ebi.ac.uk/Tools/msa/clustalw2/ [56] respectively, and presented using JalView [57]. Electrostatic potential for the P2_1_ model was calculated using Vasco [58], and quaternary structure was predicted using the PISA server http://www.ebi.ac.uk/msd-srv/prot_int/cgi-bin/piserver [59]. To calculate the buried surface area between the two P2_1_ dimers, the two chains of each dimer were merged into a single chain before uploading to PISA. PyMol [60] was used for preparing figures of the structures.

### Accession Numbers

Coordinates and structure factors have been deposited in the Protein Data Bank with accession numbers 4JZP (P3_2_21 form, acidic pH) and 4JZL (P2_1_ form, alkaline pH).

## Supporting Information

Figure S1 **IMAC pull-down of His-tagged BAP31 vDED denatured and renatured together with untagged BAP29 vDED.** L; load fraction, R; run fraction, W1-3; wash fractions, E1-3; elution fractions. The experiment was carried out by first mixing His-tagged BAP31 vDED with untagged BAP29 vDED and dialyzing against 6M guanidinium-HCl, 2 mM Tris-HCl pH 8.0 and 0.5 mM TCEP over night at room temperature. The dialysis bag was then moved into a new buffer composed of 20 mM Tris-HCl pH 8.0, 75 mM NaCl and 2 mM TCEP for renaturation. Hereafter, the sample was loaded on an open gravity flow column with 1 mL Ni-NTA beads at 4 °C. For washing, we first used 5 column volumes of renaturation buffer with 0.5 mM TCEP (W1), then 10 column volumes W1 with 20 mM imidazole (W2) and finally 10 column volumes W1 with 40 mM imidazole (W3). For elution we used W1 buffer with 200 mM imidazole in 4 times 1 column volume (E1–E4). The samples were then analyzed on SDS-PAGE.
(DOC)

Figure S2 **L-test results from Phenix Xtriage for the crystal form obtained at acidic pH.** Top, when processed in space group P622. Bottom, when processed in space group P3_2_21. Twinning is clearly present, but as no twinning is possible in P622 we could conclude that the data have lower symmetry. The correct space group was found to be P3_2_21. The only twin law possible in space group P3_2_21 is merohedral –h, –k, l. The twin fraction was estimated to be 0.436 (maximum likelihood), 0.440 (Britton analysis) or 0.447 (H-test).
(DOC)
