# Supplementary material for: Structural and Biophysical Characterization of the Cytoplasmic Domains of Human BAP29 and BAP31
Source: PLoS One. 2013 Aug 13;8(8):e71111. doi: 10.1371/journal.pone.0071111 (PMC3742741; doi:10.1371/journal.pone.0071111)
Supplement: Figure S1 — IMAC pull-down of His-tagged BAP31 vDED denatured and renatured together with untagged BAP29 vDED. L; load fraction, R; run fraction, W1-3; wash fractions, E1-3; elution fractions. The experiment was carried out by first mixing His-tagged BAP31 vDED with untagged BAP29 vDED and dialyzing against 6M guanidinium-HCl, 2 mM Tris-HCl pH 8.0 and 0.5 mM TCEP over night at room temperature. The dialysis bag was then moved into a new buffer composed of 20 mM Tris-HCl pH 8.0, 75 mM NaCl and 2 mM TCEP for renaturation. Hereafter, the sample was loaded on an open gravity flow column with 1 mL Ni-NTA beads at 4 °C. For washing, we first used 5 column volumes of renaturation buffer with 0.5 mM TCEP (W1), then 10 column volumes W1 with 20 mM imidazole (W2) and finally 10 column volumes W1 with 40 mM imidazole (W3). For elution we used W1 buffer with 200 mM imidazole in 4 times 1 column volume (E1–E4). The samples were then analyzed on SDS-PAGE. (DOC) [file pone.0071111.s001.doc]

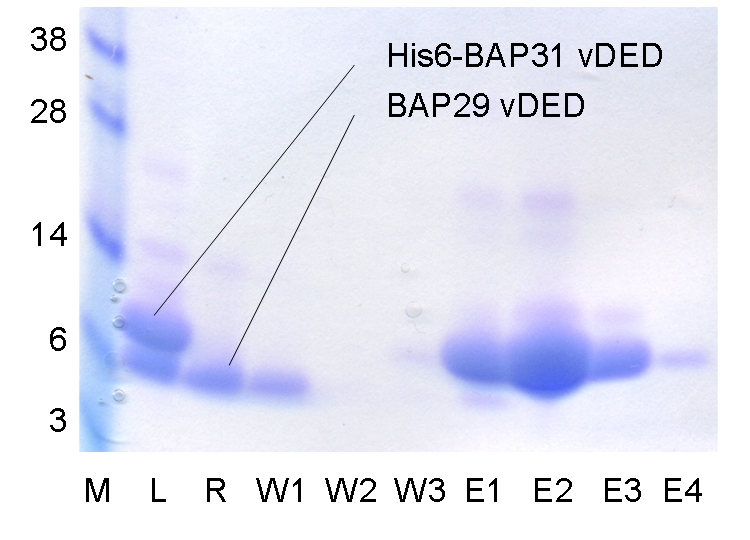


**Supplementary figure S1.** IMAC pull-down of His-tagged BAP31 vDED denatured and renatured together with untagged BAP29 vDED. L; load fraction, R; run fraction, W1-3; wash fractions, E1-3; elution fractions. The experiment was carried out by first mixing His-tagged BAP31 vDED with untagged BAP29 vDED and dialyzing against 6M guanidinium-HCl, 2 mM Tris-HCl pH 8.0 and 0.5 mM TCEP over night at room temperature. The dialysis bag was then moved into a new buffer composed of 20 mM Tris-HCl pH 8.0, 75 mM NaCl and 2 mM TCEP for renaturation. Hereafter, the sample was loaded on an open gravity flow column with 1 mL Ni-NTA beads at 4 °C. For washing, we first used 5 column volumes of renaturation buffer with 0.5 mM TCEP (W1), then 10 column volumes W1 with 20 mM imidazole (W2) and finally 10 column volumes W1 with 40 mM imidazole (W3). For elution we used W1 buffer with 200 mM imidazole in 4 times 1 column volume (E1-E4). The samples were then analyzed on SDS-PAGE.
