# Supplementary material for: Structural and Biophysical Characterization of the Cytoplasmic Domains of Human BAP29 and BAP31
Source: PLoS One. 2013 Aug 13;8(8):e71111. doi: 10.1371/journal.pone.0071111 (PMC3742741; doi:10.1371/journal.pone.0071111)
Supplement: Figure S2 — L-test results from Phenix Xtriage for the crystal form obtained at acidic pH. Top, when processed in space group P622. Bottom, when processed in space group P3221. Twinning is clearly present, but as no twinning is possible in P622 we could conclude that the data have lower symmetry. The correct space group was found to be P3221. The only twin law possible in space group P3221 is merohedral –h, –k, l. The twin fraction was estimated to be 0.436 (maximum likelihood), 0.440 (Britton analysis) or 0.447 (H-test). (DOC) [file pone.0071111.s002.doc]

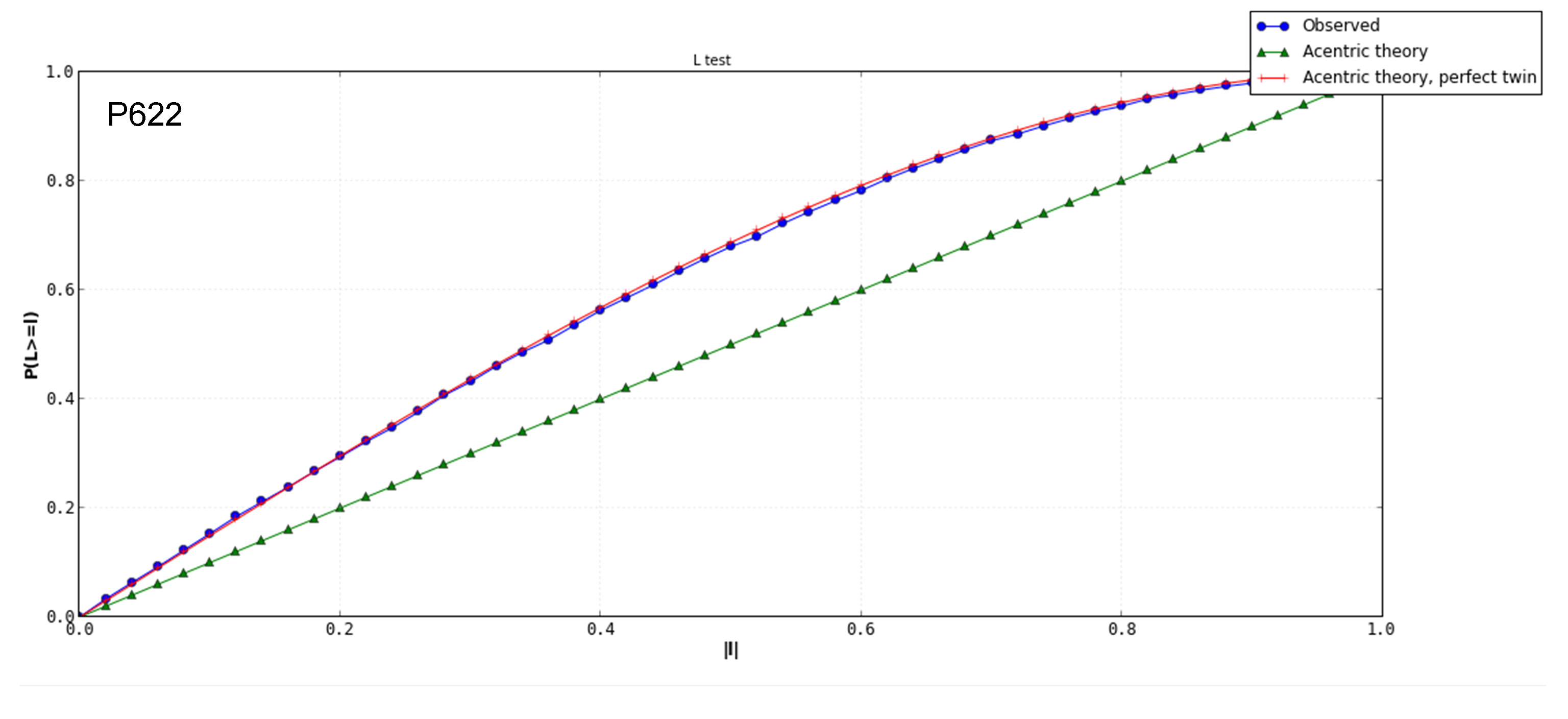


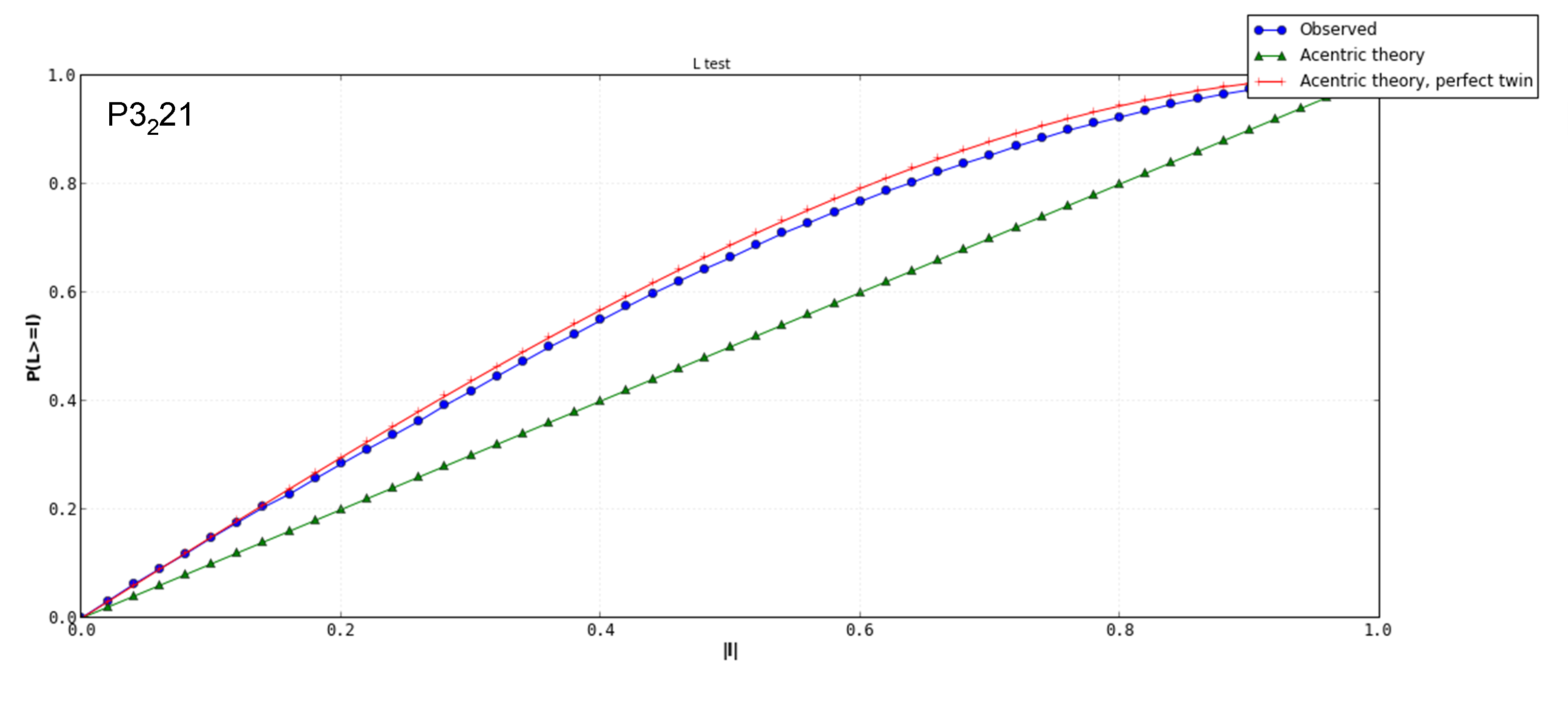


**Supplementary figure S2.** L-test results from Phenix Xtriage for the crystal form obtained at acidic pH. Top, when processed in space group P622. Bottom, when processed in space group P3_2_21. Twinning is clearly present, but as no twinning is possible in P622 we could conclude that the data have lower symmetry. The correct space group was found to be P3_2_21. The only twin law possible in space group P3_2_21 is merohedral –h, –k, l. The twin fraction was estimated to be 0.436 (maximum likelihood), 0.440 (Britton analysis) or 0.447 (H-test).
